# Mineral Composition of Wild and Cultivated Blueberries

**DOI:** 10.1007/s12011-017-1033-z

**Published:** 2017-05-09

**Authors:** Paulina Dróżdż, Vaida Šėžienė, Krystyna Pyrzynska

**Affiliations:** 1 Laboratory of Natural Environment Chemistry, Forest Research Institute, Sękocin Stary, Poland; 2 Ecology Department, Lithuanian Research Centre for Agriculture and Forestry, Kaunas distr., Lithuania; 3 Department of Chemistry, University of Warsaw, Pasteura 1, 02-093 Warsaw, Poland

**Keywords:** Blueberry, Wild growing, Cultivated, Element analysis, Water extraction

## Abstract

The concentrations of 13 elements (Al, Ca, Cd, Cr, Cu, Fe, K, Mg, Mn, Na, Ni, Pb, and Zn) were determined in several samples of native (wild) naturally growing and cultivated blueberry fruits. The total metal contents after mineralization were analyzed by inductively coupled plasma optical emission spectrometry. Reliability of the procedure was checked by the analysis of the certified reference materials Mixed Polish Herbs (INGT-MPH-2) and Leaves of Poplar (NCS DC 73350). In the fruits collected in the forest (wild blueberries), higher contents of Ca, Na, and Mg as well as Mn and Zn were observed. Similar levels of Cu, Cr, Fe, and Ni were detected in both wild-growing and cultivated plants. The significantly higher content of Fe and Cd in cultivated blueberries was connected with the content of these metals in soil samples collected from the same places. The metal extraction efficiency by hot water varied widely for the different blueberries (wild or cultivated) as well as their form (fresh or dried).

## Introduction

Among the colorful berries, *Vaccinium corymbosum*, also called American the blueberry, and the wild-growing blueberry *Vaccinium myrtillus* L. (called bilberry) are popularly used in the human diet either fresh or in processed forms. Additionally, there has been a growing trend in the use of blueberry extracts as ingredients in functional foods and dietary supplements. They are a rich source of flavonoids, phenolic acids, anthocyanins, stilbenes, and tannins, as well as nutritive compounds such as sugars, essential oils, carotenoids, vitamins, and minerals [1]. Bioactive compounds from blueberries have potent antioxidant, anticancer, antimicrobial, and anti-inflammatory properties, both in vitro and in vivo [1–3]. Blueberry fruits have been used in the traditional medicine internally (directly or as tea or liqueur) for treatment of disorders of the gastrointestinal tract and diabetes. Herbal supplements of *V. myrtillus* on the market are used for circulatory problems, as vision aids, and to treat diarrhea [4]. It was found that the consumption of wild blueberry drink for 6 weeks significantly reduced the levels of oxidized DNA bases and increased the resistance to oxidatively induced DNA damage [5].

Bilberries and American blueberries are nearly identical and used for the same purposes. Wild blueberry is found natively in Europe, northern Asia, western USA, and Canada. Unlike cultivated (highbush) blueberries, wild (lowbush) blueberries are not planted but spread primarily by rhizomes or underground runners, which give rise to new shoots and stems. Fruits are mostly collected from wild plants growing on publicly accessible lands, and you can buy them at the local markets. American blueberries, usually cultivated, grow on large bushes with the fruit in bunches and are widely available commercially.

The content of anthocyanins and other phenolic compounds in different species of blueberry (wild and cultivated) are quite often determined and compared [6–10]. Concentration of essential elements, which are also the important components of blueberry fruits, was rarely reported [11, 12]. Trace elements play an important role in the functioning of the human body. They mainly act as cofactors for various enzymatic systems (generally redox-active metals) or possess regulatory activity [13]. On the other hand, the toxic trace elements impair physiological function via the induction of universal pathogenetic pathways such as oxidative stress, inflammation, and endoplasmic reticulum stress [14].

Blueberries retain their maximum amount of nutrients and taste when they are enjoyed fresh. During the winter when fresh fruits are not available, dried blueberries can be used in the form of tea. In folk medicine, the extract from dried blueberries have been used to treat diarrhea and inflammation of the mouth or throat [4]. The study conducted by Miyake al. [15] indicated that such extract had a protective effect on visual function during retinal inflammation. The soup prepared from blueberries is also often served warm in winter or cold in summer.

The objective of this study was to investigate the level of several elements (Al, Ca, Cd, Cr, Cu, Fe, K, Mg, Mn, Na, Ni, Pb, and Zn) in native (wild) naturally growing and cultivated blueberry fruit samples. Additionally, the efficiency of extraction of these metal ions by hot water from fresh as well as dried blueberries were compared.

## Materials and Methods

### Sample Description

The fruits of wild-growing blueberries were collected during September of 2016, in three different locations in central Poland (the Mazovia region). Sample W1 was from a 12-year-old pine forest (N 52° 41′, E 21° 29′), sample W2 from a 25-year old pine forest (N 52° 49′, E 21° 45′), and sample W3 from a 77-year-old pine wood (N 52° 01′, E 21° 06′). The berries were harvested optimally ripe based on color, flavor, and structure.

Additionally, one sample (W4) was obtained from the local marketplace. One sample of cultivated American blueberries was purchased from a local market (sample C1), and the second one (sample C2) was collected from the garden plot outside Warsaw city (Poland).

Once the samples were collected, they were frozen and stored in a freezer at −20 °C. For analysis, the samples were thawed at refrigerator temperature (∼4 °C) and homogenized by blender.

### Preparation of Samples

For determination of metal contents, the samples were digested using the mixture of concentrated acids HNO_3_ and HClO_4_ (4:1 *v*/*v*). 0.5 g of fruit sample was placed into a 100-mL flask, and 10 mL of acid mixture was added to it. This mixture was heated for 30 min at 50 °C on the hot plate. Then, the temperature was slowly raised to 160–170 °C, and digestion was performed at the beginning for 120 min and finally at 200 °C for 60 min. The digested sample solution was allowed to cool down and transferred into a 50-mL volumetric flask diluted by adding distilled water. As the level of Cd and Cr in the analyzed samples was very low, additional digestion procedure was performed using higher amount of analyzed fruits (5 g).

Digestion of a reagent blank was performed in parallel.

Drying of fruits was performed in a laboratory dryer at 60 °C for 24 h.

For extraction of metals from fresh or dried blueberries, 20 mL of freshly boiled (∼95 °C) distilled water was added to 0.7 g of appropriate fruits. After 20 min of brewing and subsequent cooling, the samples were centrifuged and transferred to volumetric flasks and diluted to a final volume of 25.0 mL. The extracts were prepared directly before analysis, and all determinations were carried out in triplicate.

### Element Analysis

The concentration of elements (Al, Ca, Cd, Cr, Cu, Fe, K, Mg, Mn, Na, Ni, Pb, and Zn) were determined by inductively coupled plasma (ICP OES) using a Thermo Scientific spectrometer, model iCAP 6000. The operating parameters set to the spectrometer were those recommended by its manufacturer, i.e., the forward power of 1.15 kW, the Ar auxiliary gas flow rate of 0.2 L/min, the Ar nebulizing gas flow rate of 0.42 L/min, and coolant gas flow rate of 12 L/min. The intensity readings were repeated three times using the integration time of 1 s. The most prominent lines of the studied metals were selected, i.e., Al 396.1 nm, Ca 317.9 nm, Cd 214.4 nm, Cr 267.7 nm, Cu 224.7 nm, Fe 259.9 nm, K 766.4 nm, Mg 285.2 nm, Mn 260.5 nm, Na 589.5 nm, Ni 231.6 nm, Pb 220.3 nm, and Zn 206.2 nm.

## Results and Discussion

The total metal concentration in the digested samples of berries was determined by ICP OES method. In order to validate the analytical protocol, linearity of the calibration curve range for each element generated by injection of standard solutions as well as limit of quantification (LOQ) were evaluated. The validation data of the analytical methodology are summarized in Table 1.

**Table 1:** Validation parameters obtained for analyzed elements using ICP OES

Element	Concentration range (mg/L)	*R* ^2^	LOQ (mg/kg)	CV (%)
Al	1–20	0.9997	1.0	3.2
Ca	10–200	0.9999	0.2	1.0
Cd	0.001–0.2	0.9999	0.0003	2.6
Cr	0.05–1	1.0000	0.03	2.3
Cu	0.25–5	0.9999	0.5	1.3
Fe	0.5–10	0.9999	0.5	1.5
Mg	1–30	0.9999	2.0	1.6
Mn	2–40	0.9996	0.05	0.9
Na	1 10	0.9999	5.0	4.0
Ni	0.05–1	0.9999	0.2	4.0
Pb	0.05–1	0.9999	0.5	2.9
Zn	0.2–4	0.9999	0.3	1.0

*CV* coefficient of variation, *LOQ* limit of quantification, *R* correlation coefficient

Recovery experiments were performed for all the elements at their concentration ranges similarly found in berry fruits. The observed recoveries (data not shown) ranged between 87 and 109%, with the relative standard deviations lower than 10% in all cases. These results confirmed that no significant metal losses occurred during the digestion procedure. To verify the applicability of the proposed method, standard reference materials Mixed Polish Herbs (INGT-MPH-2) and Leaves of Poplar (NCS DC 73350) were also utilized. Digestion of these materials was performed with the same decomposition procedure used for the blueberry samples. The obtained experimental results were compared with those provided by the manufacturers and are presented in Table 2.

**Table 2:** Analysis of certified reference materials (in mg/kg, *n* = 3)

Element	Mixed Polish Herbs (INGT-MPH-2)		Leaves of Poplar (NCS DC 73350)	
	Certified	Determined	Certified	Determined
Al	670 ± 111	667 ± 10.4	1040 ± 60	1035 ± 13
Ca	10,800 ± 700	10,839 ± 176	18,100 ± 1300	181,774 ± 267
Cd	0.199 ± 0.015	0.203 ± 0.040	0.32 ± 0.07	0.32 ± 0.01
Cr	1.69 ± 0.13	1.67 ± 0.03	0.55 ± 0.07	0.55 ± 0.01
Cu	7.77 ± 0.53	7.97 ± 0.41	9.3 ± 1.0	9.23 ± 0.23
Fe	460^a^	465 ± 7.11	274 ± 17	271 ± 6.06
K	19,100 ± 120	19,135 ± 193	13,800 ± 700	13,726 ± 290
Mg	2920 ± 160	2913 ± 88.3	6500 ± 49	6549 ± 75
Mn	191 ± 12	194 ± 6.99	45 ± 4	45.0 ± 0.97
Na	350^a^	357 ± 7.09	200 ± 13	198 ± 7.11
Ni	1.57 ± 0.16	1.55 ± 0.04	1.9 ± 0.3	1.91 ± 0.05
Pb	2.16 ± 0.23	2.16 ± 0.05	1.5 ± 0.3	1.49 ± 0.08
Zn	33.5 ± 2.1	33.1 ± 0.08	37 ± 3	37.3 ± 0.56

^a^Information value

The results concerning the analyzed elements in different samples of wild and cultivated blueberry samples are presented in Table 3. All the results, expressed in milligram per kilogram on wet weight basis, are characterized by mean value with the corresponding standard deviation. Generally, in fruits collected in the forest (wild blueberries), higher levels of macroelements such as Ca, Na, and Mg (metals of natural soil origin) were observed in comparison with cultivated highbush blueberries. These minerals are dietary requirements in human nutrition and have various physiological effects. The values for Ca and Mg are at least 10-fold lower than those of potassium. This fact is common for almost all types of plants because potassium is more mobile in the xylem and phloem (vascular tissues that perform transportation of food and water in a plant) than calcium or magnesium and this element tends to be more concentrated in various parts of the plant [16]. Among microelements, higher contents of Mn and Zn were detected in naturally growing plants. Manganese is an essential element and is bound to a number of essential enzymes, for example, the activity of superoxide dismutase is suppressed by low Mn status [17]. Similar levels of Cu, Cr, Fe, and Ni were detected in both wild-growing and cultivated plants.

**Table 3:** Content of elements in different blueberry samples in mg/kg (mean ± SD, *n* = 3)

Sample	Al	Ca	Cd	Cr	Cu	Fe	K
W1	14.8 ± 0.55a	185 ± 9.7a	0.004 ± 0.0003a	0.039 ± 0.002a	0.31 ± 0.017a	8.38 ± 0.33a	880 ± 27a
W2	12.4 ± 0.60b	164 ± 7.9b	0.002 ± 0.0001b	0.174 ± 0.008b	0.26 ± 0.012b	7.82 ± 0.39a	1036 ± 51b
W3	15.1 ± 0.67a	203 ± 8.9ac	0.001 ± 0.0005c	0.023 ± 0.001c	0.24 ± 0.013c	5.31 ± 0.25b	1090 ± 52b
W4	16.3 ± 0.73a	192 ± 8.7ac	0.003 ± 0.0001d	0.084 ± 0.004d	0.41 ± 0.016d	12.7 ± 0.64c	1105 ± 53b
C1	13.3 ± 0.91	112 ± 5.1d	0.013 ± 0.0006e	0.033 ± 0.006e	0.28 ± 0.011abce	5.44 ± 0.27d	825 ± 15a
C2	17.9 ± 0.80	162 ± 6.1b	0.040 ± 0.0015f	0.014 ± 0.001e	0.32 ± 0.014ae	10.7 ± 0.52d	1031 ± 28b
	Mg	Mn	Na	Ni	Pb	Zn	
W1	84.2 ± 6.92a	17.1 ± 1.03a	52.7 ± 1.93a	0.263 ± 0.019a	0.556 ± 0.063a	6.07 ± 0.75a	
W2	75.1 ± 3.09a	5.82 ± 0.16b	54.0 ± 1.78a	0.254 ± 0.012a	0.373 ± 0.018b	5.26 ± 0.26ab	
W3	96.9 ± 2.11b	29.0 ± 1.35c	54.9 ± 2.03a	0.252 ± 0.011a	0.266 ± 0.013b	4.22 ± 0.17bcd	
W4	90.1 ± 3.42b	18.5 ± 0.88a	61.6 ± 2.97b	0.312 ± 0.015b	0.396 ± 0.018ab	3.83 ± 0.15c	
C1	51.4 ± 2.60c	4.47 ± 0.13d	48.1 ± 2.12c	0.170 ± 0.008a	0.276 ± 0.014b	2.95 ± 0.15ce	
C2	48.1 ± 2.07c	2.94 ± 0.015e	49.2 ± 2.63c	0.299 ± 0.015a	0.315 ± 0.015b	3.79 ± 0.19cd	

Values followed by the same letters in each column (a, b, c, d) have no significant difference (Tukey’s test, *p* > 0.05).

*W* stands for wild blueberry and *C* stands for cultivated American blueberry (see experimental).

Significantly higher content of cadmium was detected in fruits of farm-raised in comparison to wild cultivars. It can be explained by the much higher Cd content in the soil sample collected from the same place (0.398 mg/kg) in comparison with 0.041–0.087 mg/kg determined in soils, where W1–W4 samples were grown. According to The Commission of the European Communities [18], the maximum allowed levels for Cd and Pb are 0.05 and 0.2 mg/kg, respectively, which are higher than the values detected in this study. Hence, none of these berries would represent a risk to human health from this point of view. Vollmannova et al. [11] found higher Cu, Zn, and Pb and similar Cd contents in wild blueberries collected in Slovakia in comparison with highbush blueberry cultivar; however, the soil content where the fruits were grown was not examined.

In the present study, the Fe concentration in cultivated blueberries is higher than that in other reports [12, 16], probably due to soil conditions. The sample was collected from the garden plot where acidic soil with high content of Fe (4260 mg/kg) as well as Al (3000 mg/kg) dominates. A similar behavior for iron in different berry fruits cultivated in Brazil has been reported [19]. That fact can justify our results.

Considering the content of individual elements, statistically significant differences were observed among the wild-growing blueberries collected in different locations (samples W1–W4). These variations were probably due to different growing conditions, soil load, and soil properties as well as periodic changes in pollution of soil, water, and air [20]. Although, we selected a limited area for sample collection in order to have uniform climatic conditions. The results of one-way ANOVA followed by Tukey’s test indicated that the contents of Cd, Cr, Cu, and Mn vary significantly (*p* < 0.05) among all four studied samples.

**Fig. 1:**
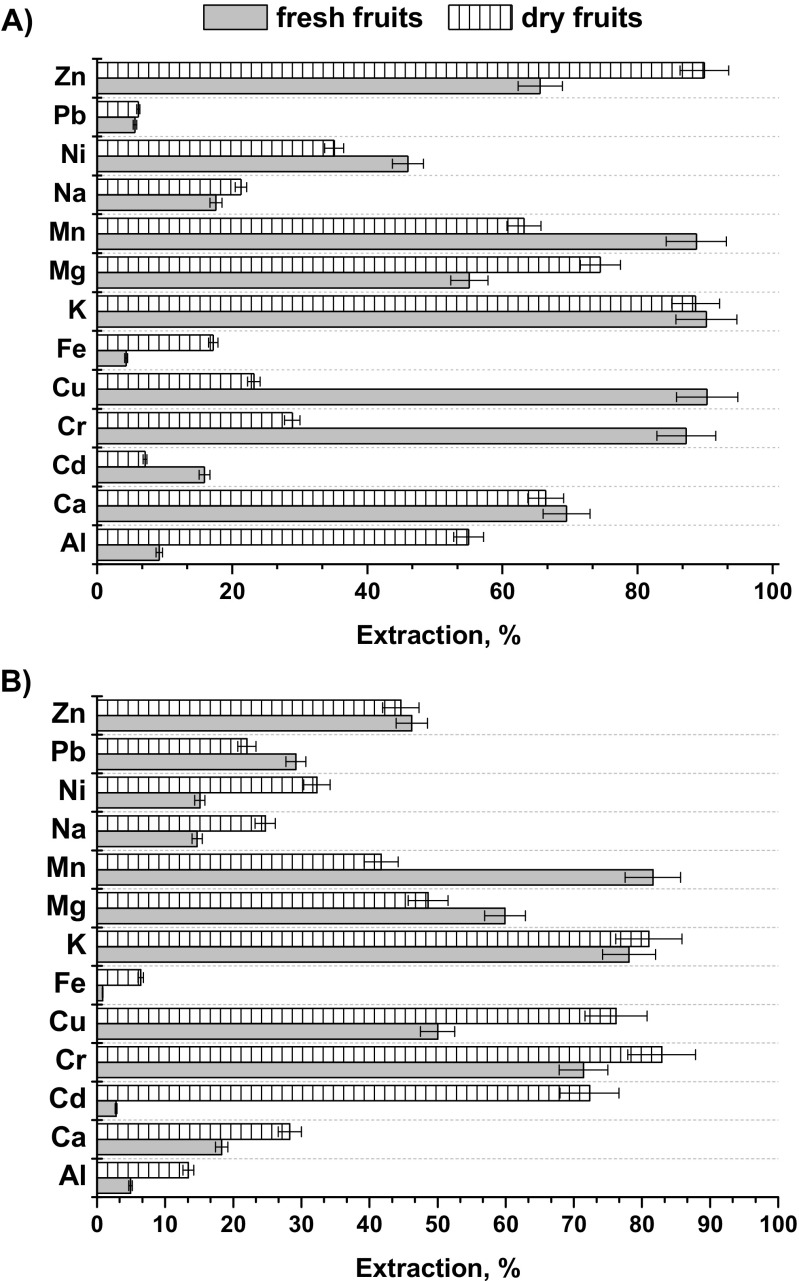
Efficiency of extraction of metal ions from **a** wild blueberries (sample W1) and **b** cultivated blueberries (sample C2). Fresh fruits, dry fruits

Blueberries, in fresh or dry form, can be also used for preparation of hot or cold beverages such as tea, juice, or syrup. Thus, it was interesting to check the efficiency of extraction of metal ions by hot water from the fresh as well as from the dried blueberry fruits under study. Samples W1 and C2 (wild and cultivated berries) were chosen for these experiments. Drying of these fruits was performed at 60 °C for 24 h, and acidic digestion was then performed with the same procedure as for fresh fruits. Extraction of metals was performed using 20 mL of freshly boiled distilled water for 20 min. Concentration of metals in the obtained infusions was determined by ICP OES method. The obtained results are presented in Fig. 1. The metal extraction efficiency varied widely for the different blueberries (wild or cultivated) as well as their form (fresh or dried). Generally, the percent of extraction exceeded 80% for K, Cr, and Cu (only for fresh fruits) from all types of fruits. Also the efficiency of extraction for Mn from both wild blueberries and Zn from fresh fruits are very high. The low extractability of Fe was also reported from different kinds of tea [21]. However, the bioaccessibility of this element is greater than that in the other berry fruits [16]. Interesting, the leaching of toxic elements such as Pb and Cd from fresh and dried wild blueberries was very little.

## Conclusion

The mineral composition of wild and cultivated blueberries has been determined by ICP OES after mineralization. Forest (wild blueberries) proved to be good sources of Ca, Na, and Mg as well as Mn and Zn. Mn is also extracted with hot water either from fresh or dried wild blueberries to a great extent. Moreover, only low contents of toxic elements such as Pb and Cd were detected, ensuring the absence of risk for human health.
